# Hypoglutamatergic state is associated with reduced cerebral glucose metabolism in anti-NMDA receptor encephalitis: a case report

**DOI:** 10.1186/s12888-015-0552-4

**Published:** 2015-08-01

**Authors:** Dominique Endres, Evgeniy Perlov, Oliver Stich, Sebastian Rauer, Simon Maier, Zora Waldkircher, Thomas Lange, Irina Mader, Philipp Tobias Meyer, Ludger Tebartz van Elst

**Affiliations:** Section of Experimental Neuropsychiatry, Department for Psychiatry& Psychotherapy, University Medical Center Freiburg, Hauptstr. 5, 79104 Freiburg, Germany; Freiburg Brain Imaging, University Medical Center Freiburg, Breisacher Str. 64, 79106 Freiburg, Germany; Department of Neurology, University Medical Center Freiburg, Breisacher Str. 64, 79106 Freiburg, Germany; Department of Radiology, Medical Physics, University Medical Center Freiburg, Breisacher Str. 64, 79106 Freiburg, Germany; Department of Neuroradiology, University Medical Center Freiburg, Breisacher Str. 64, 79106 Freiburg, Germany; Department of Nuclear Medicine, University Medical Center Freiburg, Hugstetter Str. 64, 79106 Freiburg, Germany

**Keywords:** NMDA-receptor, Anti-NMDA-receptor-encephalitis, Glutamate, Magnetic resonance spectroscopy, Fluorodeoxyglucose positron emission tomography

## Abstract

**Background:**

Anti-N-methyl-D-aspartate receptor (NMDAR) encephalitis was first described in 2005 in association with ovarian teratoma. The diagnostic workup of this common autoimmune encephalitis includes cerebrospinal fluid analysis, electroencephalography, magnetic resonance imaging (MRI), and fluorodeoxyglucose positron emission tomography (FDG-PET). In addition to standard diagnostics, we performed metabolic investigations using proton magnet resonance spectroscopy (^1^H-MRS).

**Case presentation:**

We describe the case of a non-limbic anti-NMDAR encephalitis with a long course of disease (21 months). Laboratory diagnostics showed antibodies against NMDAR; an MRI revealed unspecific findings. ^1^H-MRS indicated a hypoglutamatergic state in the left prefrontal cortex associated with a left hemispherical hypometabolism on FDG-PET. Despite the long course of disease, immunosuppressive therapy with methylprednisolone and azathioprine led to almost complete remission of clinical symptoms. Clinical improvement developed in parallel with remarkable normalization of cerebral glucose metabolism on FDG-PET.

**Conclusion:**

This case of long-lasting extra-limbic anti-NMDAR encephalitis is of high clinical relevance. First, it illustrates that a very good outcome is possible even if adequate therapy is started only 21 months after the onset of severe symptoms. Second, it provides valuable insights into the pathophysiology of such anti-NMDAR encephalitis; these insights prove that anti-NMDAR encephalitis is linked not only to hyperglutamatergic signals but also to hypoglutamatergic states. These findings, contradictory at first glance, can be integrated within the model of excitatory/inhibitory imbalance and local area network inhibition.

## Background

Immunological encephalopathies (IE) are increasingly recognized in psychiatry as rare but still important causes of clinical syndromes, which often present as atypical psychoses or affective disorders. IE may also present as a classical affective or psychotic syndrome without the hallmarks of organic causes. In this paper, we want to illustrate this new and complex clinical issue with respect to anti-N-methyl-D-aspartate receptor (NMDAR) encephalitis by presenting a remarkable, severe, and chronic case of IE with positive outcome.

**Table 1:** Magnetic resonance spectroscopy (^1^H-MRS) results

	Left prefrontal cortex		Right prefrontal cortex	
	Metabolite concentration (in IU) (CRLB)	Metabolite ratio (/Cre)	Metabolite concentration (in IU) (CRLB)	Metabolite ratio(/Cre)
Glx	5.673 (12 %)	1.129	7.424 (8 %)	1.576
Cre	5.023 (3 %)	1.0	4.710 (3 %)	1.0
t-Cho	1.238 (3 %)	0.246	1.217 (3 %)	0.258
NAA + NAAG	6.783 (3 %)	1.350	5.802 (3 %)	1.232
mI	3.815 (5 %)	0.760	3.295 (5 %)	0.699

The MRS spectrum from the left prefrontal cortex shows lower concentrations of Glx compared with the opposite side. Glutamatergic hypometabolism correlated with a distinct hypometabolism in FDG-PET in this region; TR = 3000; TE = 30; NS = 64; voxel size = 8.0 mL. Abbreviations: CRLB = Cramér-Rao lower bounds; IU = Institutional Units; Glx = glutamate + glutamate; Cre = creatine; t-Cho = phosphorylcholine + glycerophosphorylcholine; NAA + NAAG = N-acetylaspartate + N-acetyl-aspartyl glutamate; mI = myo-inositol

### Anti-NMDA receptor encephalitis

The anti-NMDAR encephalitis was first described in 2005 in association with ovarian teratoma [1, 2], and was followed by an still increasing number of case reports and case series. In 2013, Titulaer and colleagues described 577 patients in the hitherto existing largest cohort study [3]. Some authors claim that anti-NMDAR encephalitis is the second most frequent autoimmune encephalitis, after acute demyelinating encephalomyelitis [4].

Pathophysiologically, the initiation of anti-NMDAR-antibody production has yet to be understood in detail. In accordance with current theories, lymphocyte production is stimulated by a peripheral initiator, such as a tumor or infection. The disruption of the blood–brain barrier allows the passage of immune cells into the central nervous system (CNS) and leads to the clonal expansion of lymphocyte populations in the CNS, resulting in intrathecal antibody production [5]. Antibody binding to the NR1 subunit of the NMDA receptor leads to the internalization of the NMDA receptor via a cross-linking process with anti-Fab antibodies [6–8]. Internalization creates a reversible NMDAR hypofunction without the destruction of neurons or synapses [7, 9].

The clinical course of anti-NMDAR encephalitis is characterized by different phases of the disease: 1) prodromal period with headache, fever, or nausea; 2) psychiatric period with anxiety, paranoia, delusions, short-term memory loss, disintegration of language and sometimes mutism; 3) reduced consciousness; 4) hypoventilation; 5) seizures; 6) autonomic instability with, for example, hyperthermia, tachycardia, or urinary incontinence and dyskinesia; and 7) recovery in approximately 75 % or death [8, 10, 11]. In 60 % of patients, anti-NMDAR encephalitis is paraneoplastic, most often associated with ovarian teratoma [10]. The diagnostic workup includes cerebrospinal fluid (CSF) analysis, electroencephalography (EEG) and magnetic resonance imaging (MRI). Common differential diagnosis (especially infectious ones) should be clarified, and tumor screening should always be included in the diagnostic work up. The CSF examination shows initial abnormalities in 80 % of patients; protein concentration and white blood cell (WBC) counts are generally increased in a moderate way. CSF specific oligoclonal bands can be found in 60 % of patients. An intrathecal synthesis of anti-NMDA receptor antibodies is the most specific indicator [8]. EEG is abnormal in over 90 % of patients, and often shows diffuse slow activity [8, 10]. In 30 % of patients, a unique electrographic pattern called “extreme delta brush” was observed [12, 13]. In 50 % of the cases, the MRI has no pathological findings, while T2 or FLAIR hyperintensity is found in different regions in the remaining 50 % of cases [8]. Some studies showed abnormalities on fluorodeoxyglucose positron emission tomography (FDG-PET) or single-photon emission computed tomography [14–16].

Proton magnetic resonance spectroscopy (^1^H-MRS) might be another diagnostic tool to investigate anti-NMDAR encephalitis by measuring absolute concentrations of glutamate (Glu), glutamine (Gln), and the combined Glu and Gln signal, which is abbreviated as Glx.

Although randomized trials for the treatment of anti-NMDAR encephalitis are lacking, high-dose intravenous corticosteroids, plasma exchange, intravenous immunoglobulins, azathioprine and monoclonal antibodies (e.g., rituximab) are commonly used [17].

### Neurometabolic imaging

The FDG-PET visualizes regional neuronal activity by measuring cerebral glucose (Glc) metabolism. However, only ^1^H-MRS allows non-invasive and non-radioactive measurement of Glu metabolism. ^1^H-MRS is a MR technique, that takes advantage of the fact that the resonance frequency of certain molecules reflects their structure and, thereby, their identity. ^1^H-MRS allows the absolute quantification of Glx, phosphorylcholine and glycerophosphorylcholine (t-Cho), N-acetylaspartate (NAA), creatine (Cre) and myo-inositol (mI). Glu is the major excitatory neurotransmitter in the human brain [18], while Gln is its storage and precursor form in astrocytes [19]. NAA is a marker of neuronal and axonal integrity. t-Cho is a marker for cell membrane turnover. Cre is often used as a concentration reference substance based on its constancy with respect to many pathologies. Finally, ^1^H-MRS allows the detection of mI, which is seen as a glial marker and part of the phosphatidyl-inositol second messenger system [20].

## Case presentation

### Specific characteristics

We present the case of a patient with an anti-NMDAR encephalitis, with a left prefrontal hypoglutamatergic status as measured with single-voxel ^1^H-MRS associated with a left hemispheric hypometabolism shown by FDG-PET. Despite the long duration of the disease, immunosuppressive therapy was successful.

### Medical history

The patient is a 31-year-old woman who had worked successfully as a business controller for years. Twenty-one months prior to final diagnosis (day 0, onset), she experienced the first signs of personality change for approximately two weeks (mood changes and compulsive behaviors). Two months later, she experienced an epileptic seizure (for the first time), affective destabilization, cognitive deficits, and aggressiveness. Examination of the CSF showed an increased WBC count (23 /μl) and an elevated protein concentration (487 mg/l). No infectious cause was identified, and the MRI showed no pathological findings. Treatment was initiated with ceftriaxone and aciclovir for possible pathogenic agents, and clobazam and valproate for seizures. Despite treatment, the patient developed increasing mood changes, disorganized agitated behavior, partial amnesia, disinhibition, and ongoing aggressiveness. Additionally, she developed delusional symptoms and delirious states, which were treated with antipsychotic medication and benzodiazepines, as several physicians involved in the case management preferred the diagnosis of schizophrenia. One month later (~day 90, onset), the patient developed a catatonic state with distinctive waxy flexibility and no reaction to strong pain stimuli. At that time, the diagnosis of catatonic schizophrenia was made. Two months later (~day 150, onset), the condition had improved, and the patient presented with a loss of energy, anxiety, and decelerated thinking, but without delusions, hallucinations or catatonic states. No further antipsychotic therapy was prescribed. Nearly one year later (~day 500, onset), the patient attended a neurological rehabilitation center. At that time, she still presented with loss of energy and pronounced difficulties in concentration. Furthermore, at that time, she was completely withdrawn socially. Because of psychotic and epileptic exacerbations, the diagnostic investigations were repeated (~day 540, onset). WBC count (20/μl) and protein concentration (550 mg/l) in the CSF were still elevated. A long-term EEG examination revealed a left temporal and right hemispherical brain dysfunction without epileptiform patterns. In the MRI, right temporo*‐*occipital signal alterations were detected. A treatment with aciclovir, with the addition of piperacillin, was again unsuccessful. Psychotic symptoms increased and were treated again with antipsychotic drugs and high doses of tranquilizers. Meanwhile, anticonvulsive medication was changed to oxcarbazepine and lamotrigine. Neither medication was tolerated, due to leukopenia, thrombopenia, and exanthema; therefore, monotherapy was continued with valproate. The state of health deteriorated progressively with enuresis and an inability to eat, drink, and carry out personal hygiene. Twenty months after the first symptoms, the patient was admitted to the university clinic of psychiatry & psychotherapy Freiburg for further investigations.

### Clinical presentation

On admission, the patient presented in a tired but fully conscious and oriented status. She showed difficulties in concentration, loss of memory, and disturbed speech production. On the first day, the patient already showed states of altered consciousness, mutism, and bizarre behavior. She answered questions with laughing, repeated single syllables (echolalia, palilalia), displayed fidgeting movements (right hand), showed ocular muscle contractions (left), was smacking her lips and displaying stereotypical movements. The patient was repeatedly frozen and showed symptoms of waxy flexibility.

### Diagnostic results

**Fig. 1:**
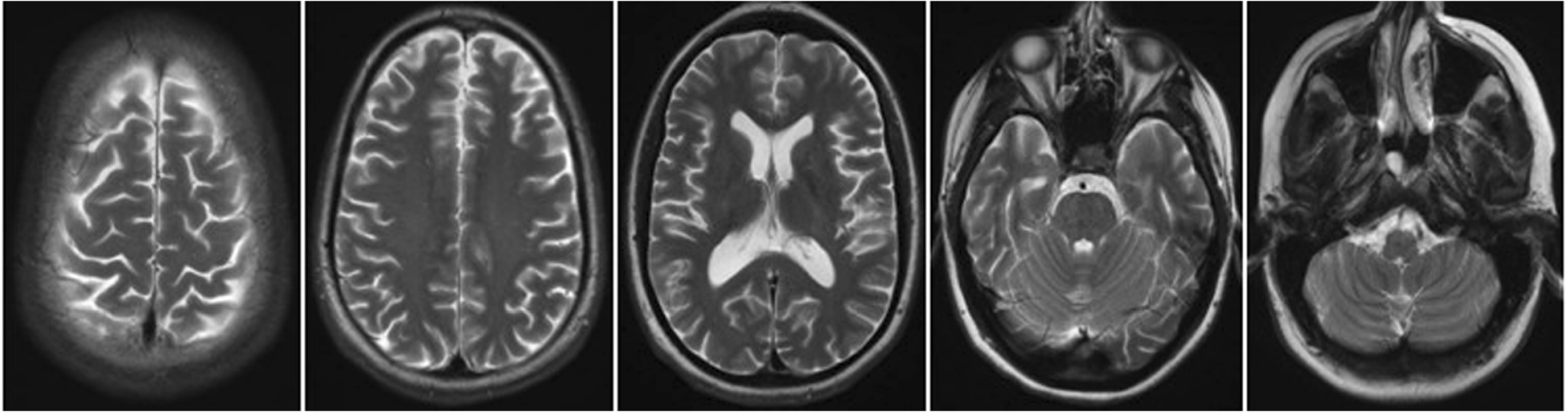
Magnet resonance imaging (MRI) findings. MRI only shows a moderate perisylvic/temporal accentuated atrophy

**Fig. 2:**
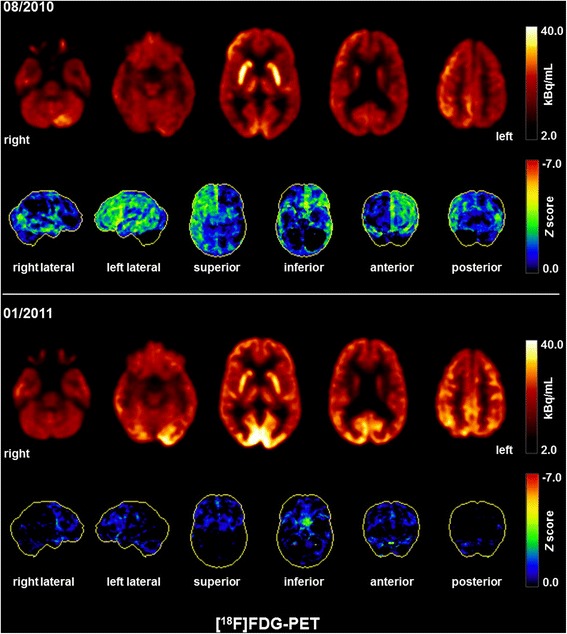
Fluorodeoxyglucose positron emission tomography (FDG-PET) findings. The initial FDG-PET scan (08/2010) depicted a global cortical hypometabolism of the left hemisphere, and, to a lesser extent, also of the right hemisphere; on the right side, hypometabolism was primarily located in the temporal lobe. Cerebellar hypometabolism was pronounced on the right side (most likely indicating crossed cerebellar diaschisis). The lower FDG-PET scan was acquired after successful immunosuppressive therapy (01/2011), indicating a nearly regular cerebral metabolism. A slight frontal hypometabolism remained most likely secondary due to frontal accentuated atrophy. Pronounced occipital metabolism is explainable by scan acquisition with open eyes. Both FDG-PET scans were performed at the Department of Nuclear Medicine of the University Hospital Freiburg after injection of 240 MBq 18FDG each (Gemini TF64 PET scanner, Philips Healthcare, The Netherlands)

In the CSF analysis, we found a blood–brain barrier disturbance (protein: 561 mg/l, albumin-quotient: 8.7), normal cell count (1/μl), and no intrathecal immunoglobulin synthesis. Serological screening for antibodies against neuronal cell surface antigens showed antibodies against the NMDA receptor. The analysis was performed in the reference laboratory at the Weatherall Institute of Molecular Medicine at John Radcliffe Hospital (Oxford, UK). EEG analysis showed an intermittent delta focus over the right central areas. In an additional independent component analysis of the EEG, we were able to describe three components: 1) right and left frontotemporal delta waves; 2) a deep right temporal generator; and 3) a central component including theta frequencies. MRI showed no specific findings; especially the hippocampal regions and structures of limbic system were without pathological findings. Moderate perisylvic/temporal accentuated atrophy was found (Fig. 1). On FDG-PET, global cortical hypometabolism of the left hemisphere was detected. A less pronounced hypometabolism was also detected on the right side, particularly of the temporal lobe. Cerebellar hypometabolism was found predominantly on the right side (most likely indicating crossed cerebellar diaschisis) (Fig. 2). ^1^H-MRS was performed in the prefrontal cortex (PFC) on both sides, using the standard single-voxel spectroscopy (voxel size 8 ml) Table 1. For spectroscopic analysis, the well-established and investigator-independent LCModel (linear combination of model spectra) algorithm was used (www.s-provencher.com/pages/lcmodel.shtml) [21, 22]. In the PFC of the hypometabolic left hemisphere, we found distinct lower Glx concentrations compared with the opposite side. Glx/Cr ratios were also significantly decreased.

### Therapeutic and clinical course

A diagnosis of anti-NMDAR encephalitis was established nearly 21 months after onset on the basis of typical clinical symptoms and the serological detection of antibodies against the NMDA receptor. We started an initial treatment with plasma exchange over 2 weeks (5 sessions) and prednisone (70 mg/d). However, this treatment did not lead to any improvement; therefore, after 10 days, we began a high-dose steroid pulse therapy (methylprednisolone 500 mg i.v./day for 5 days) and subsequently, a low-dose oral cortisone tapering until replacement with azathioprine as chronic maintenance therapy. Additionally, the antiepileptic medication was changed from valproate to levetiracetam. During and after the steroid pulse therapy, daily improvement was visible. Language production normalized and mood swings and disorganized behavior disappeared. The patient was discharged from the psychiatric department to the neuropsychiatric rehabilitation.

A second FDG-PET five months after the start of steroid treatment (~day 790, onset) indicated a nearly regular metabolism (Fig. 2). A slight frontal hypometabolism remained, probably due to frontal accentuated atrophy. In cognitive testing before discharge, slight deficits, particularly in working memory and mental flexibility, were still present. The repeated gynecological tumor screening for teratoma showed no pathological findings up to date.

## Conclusion

### Characteristics of our case report

We present the case of a 31-year-old woman with severe neuropsychiatric symptoms which were classified as schizophrenia or catatonic schizophrenia by several clinicians involved in case management but who in fact was suffering from an anti-NMDAR encephalitis. Neurometabolic investigations showed a left prefrontal hypoglutamatergic status in ^1^H-MRS associated with a left hemispheric hypometabolism on FDG-PET. To our knowledge, this is the first report of cortical hypoglutamatergic in vivo metabolism in anti-NMDAR encephalitis, illustrating that this mechanism might be of pathogenetic relevance in the genesis of psychiatric symptoms in such constellations. Despite the long duration of the neuroinflammatory process (21 months), immunosuppressive therapy was still successful. Clinical improvement paralleled the normalization of the metabolism on FDG-PET.

### FDG-PET in autoimmune encephalitis

FDG-PET provides greater sensitivity for autoimmune encephalitis (78 %) compared with MRI (63 %) [23]. In our case report, we observed non-specific changes with MRI and remarkable abnormalities with the FDG-PET. In a retrospective analysis of FDG-PET findings with regard to autoimmune encephalitis, a significant association between autoantibody type and PET findings was described: in patients with autoantibodies against intracellular antigens, mainly mesiotemporal abnormalities were found, whereas normal findings or abnormalities outside the mesiotemporal region were more often detected in patients with autoantibodies against surface antigens, such as antibodies against the NMDA receptor [23]. Specifically, patients with anti-NMDAR encephalitis showed frontal and temporal Glc hypermetabolism associated with occipital hypometabolism (frontotemporal-to-occipital gradient). This pattern was positively associated with clinical disease severity. On longitudinal measurements of two patients, a normalization of cerebral Glc metabolism was identified [24]. We detected deviating FDG-PET results; in our case study, a global cortical hypometabolism was found in the left hemisphere, and, to a lesser degree, also in the right hemisphere. Moreover, cerebellar hypometabolism was identified, particularly on the right hemisphere, likely secondary to crossed cerebellar diaschisis. In our case, cortical hypometabolism might be the consequence of the long-term process which became apparent on FDG-PET 21 months after onset of the disease. By contrast, hypermetabolism in the frontotemporal regions might be an indicator of an acute disease phase [15], as Glc metabolism increases in active inflammation [25]. Consequently, in the study from Leypoldt and colleagues showing frontotemporal hypermetabolism, there was a distinctly shorter median time (median time was 10 weeks; range from 10 to 30 weeks) from the onset of the disease until FDG-PET imaging [24]. However, it is important to note that cortical hypometabolism did not indicate irreversible neuronal injury or post-inflammatory residuum in our case, because metabolism largely recovered upon treatment in parallel to clinical improvement.

### ^1^H-MRS in autoimmune encephalitis

To our knowledge, only two ^1^H-MRS measurements in anti-NMDAR encephalitis have been published to date [26, 27]. In the first case, NAA signals were initially decreased and normalized in the clinical course [26]. In the second case, reduced NAA concentrations were observed in the basal ganglia and thalamus at an early stage. Despite reduced NAA levels, MRI and EEG findings were unremarkable in this case. In late stage and after resolution of involuntary movements, NAA signals normalized [27]. In our case, we only detected a tendency towards higher NAA concentrations in the hypometabolic left hemisphere. However, we found evidence of decreased Glx signals and Glx/Cre ratios in the hypometabolic left hemisphere compared with the right side. It is tempting to speculate that this underlies the observed, reversible hypometabolism on FDG-PET, which may be due to a persistent NMDAR hypofunctional state after the initial acute inflammatory and hypermetabolic phase observed by others. The reversibility of this process is likely the reason for the good prognosis of our patient, even though the pathogenic process went untreated for nearly 21 months. The decrease of NMDA receptors directly correlates with the antibody titers [8]. Manto and colleagues performed *in vivo* experiments using Glu. They were able to identify increased Glu concentrations in the extracellular space. The increase was dose-dependent and much more pronounced with purified IgG [28].

### Excitatory/inhibitory dysbalance and local area network inhibition

The findings of extracellular hyperglutamatergic states reported by Manto et al. [28] and our findings of hypoglutamatergic signal in ^1^H-MRS seem to be contradictory at first glance. However, they might well be integrated within the theoretical framework of excitatory/inhibitory dysbalance and local area network inhibition [29–31]. Similar results indicating a change from hyper- to hypoglutamatergic status are well known in epilepsy research. Excitatory seizure activity initially leads to an acute increase in Glx concentrations and a subsequent decrease in these concentrations over the course of the disease [29, 30, 32, 33]. A state of neuronal network instability as seen in epilepsy can be associated with local area hyperexcitability and hyperinhibition [31]. Therefore, we speculate that a model of a neuronal excitatory/inhibitory imbalance might explain the plethora of neuropsychiatric symptoms that can be observed in the course of NMDR encephalitis: Initial hyperglutamatergic states can be interpreted as an indicator of cortical over-excitation, whereas hypoglutamatergic states in the further course (after months) of the disease might be a sequel of over-inhibition of cortical brain areas, possibly triggered by the preceding over-excitation. We have recently put forward a model of local area network inhibition (LANI hypothesis) that is capable of explaining how states of excitatory/inhibitory dysbalances might translate pathogenetically into a plethora of neuropsychiatric symptoms typically seen in “organic psychiatric disorders” [31]. Such a model is also capable of explaining the symptoms of our patient. Initial and intercurrent hyperglutamatergic states might be linked to her hyperexcitability and symptoms such as seizures, whereas hypoglutamatergic states might be the cause for hyperinhibition and symptoms like her mutistic and delirious states.

### Neuroinflammation and glutamatergic metabolism

Furthermore, it is important to recognize that neuroinflammation is itself associated with altered glutamatergic metabolism. For example, the reduction of extracellular glutamate concentrations, through the intake of Glu into astrocytes, is disturbed during the inflammatory process [34]. Disturbed glutamate signals have also been shown to occur in neuroinflammatory diseases, such as HIV encephalopathy [35]. Thus, it seems reasonable to combine Glc (FDG-PET) and Glu (^1^H-MRS) measurements in cases in which anti-NMDAR encephalitis is suspected.

### Summary

We presented the case of a 31-year-old woman with anti-NMDAR encephalitis. Neurometabolic investigations showed a left prefrontal hypoglutamatergic status on ^1^H-MRS associated with left hemispheric hypometabolism on FDG-PET. This case is remarkable because, to our knowledge, this is the first report on in vivo hypoglutamatergic status in anti-NMDAR encephalitis. The non-invasive measurement of the Glx signals via ^1^H-MRS might directly provide insights into the pathomechanism of anti-NMDAR encephalitis. In further studies, the combination of EEG, MRI, FDG-PET, and ^1^H-MRS should be performed during different stages of disease progression. Finally—and, clinically, even more important—our case report illustrates that even a retarded initiation of immunosuppressive therapy might be effective, and could lead to nearly full clinical remission.

## Consent

The patient has given her consent for the details of the case report and for the figures to be published.
